# Extracellular Vesicles and Transforming Growth Factor β Signaling in Cancer

**DOI:** 10.3389/fcell.2022.849938

**Published:** 2022-04-13

**Authors:** Dorival Mendes Rodrigues-Junior, Chrysoula Tsirigoti, Sai Kiang Lim, Carl-Henrik Heldin, Aristidis Moustakas

**Affiliations:** ^1^ Department of Medical Biochemistry and Microbiology, Uppsala University, Uppsala, Sweden; ^2^ Institute of Molecular and Cell Biology (A*-STAR), Singapore, Singapore

**Keywords:** cancer-associated fibroblast (CAF), epithelial – mesenchymal transition (EMT), extracellular vesicle (EV), metastasis, micro-RNA (miRNA), transforming growth factor β (TGF-β)

## Abstract

Complexity in mechanisms that drive cancer development and progression is exemplified by the transforming growth factor β (TGF-β) signaling pathway, which suppresses early-stage hyperplasia, yet assists aggressive tumors to achieve metastasis. Of note, several molecules, including mRNAs, non-coding RNAs, and proteins known to be associated with the TGF-β pathway have been reported as constituents in the cargo of extracellular vesicles (EVs). EVs are secreted vesicles delimited by a lipid bilayer and play critical functions in intercellular communication, including regulation of the tumor microenvironment and cancer development. Thus, this review aims at summarizing the impact of EVs on TGF-β signaling by focusing on mechanisms by which EV cargo can influence tumorigenesis, metastatic spread, immune evasion and response to anti-cancer treatment. Moreover, we emphasize the potential of TGF-β-related molecules present in circulating EVs as useful biomarkers of prognosis, diagnosis, and prediction of response to treatment in cancer patients.

## Introduction

Extracellular vesicles (EVs) is an ISEV (International Society for Extracellular Vesicles)-endorsed, collective term for nanosized lipid membrane vesicles that are naturally released from cells (Théry et al., 2018). EVs are heterogeneous and are subtyped according to their biogenesis. Identifying or isolating the various EV subtypes is challenging as no definitive markers can discriminate between subtypes with high security. Accordingly, ISEV recommends the use of operational terms to differentiate various experimentally-obtained EV populations, such as small EVs (sEVs; EVs with a diameter <100 nm). The function of EVs is to mediate intercellular communication in physiological or pathological processes by trafficking biologically active molecules (proteins, nucleic acids, lipids and carbohydrates) from secreting to recipient cells, and even to remote sites *via* circulation through bodily fluids such as the blood (Raposo and Stoorvogel, 2013). EVs interact with recipient cells in several ways. They can be internalized by recipient cells *via* a membrane to membrane fusion process or *via* endocytosis that shuttles them to endocytic compartments where the cargo is released into the cytoplasm or shuttled to lysosomes for degradation (Raposo and Stoorvogel, 2013). Alternatively, EVs could exert their effects on cells without being internalized through various membrane proteins such as CD73, CD59 and others, as explained later. Certain EV membrane proteins could hydrolyze extracellular AMP to activate receptor-mediated signaling or inhibit complement complex formation and prevent cell lysis, respectively (Lai et al., 2012).

**FIGURE 1:**
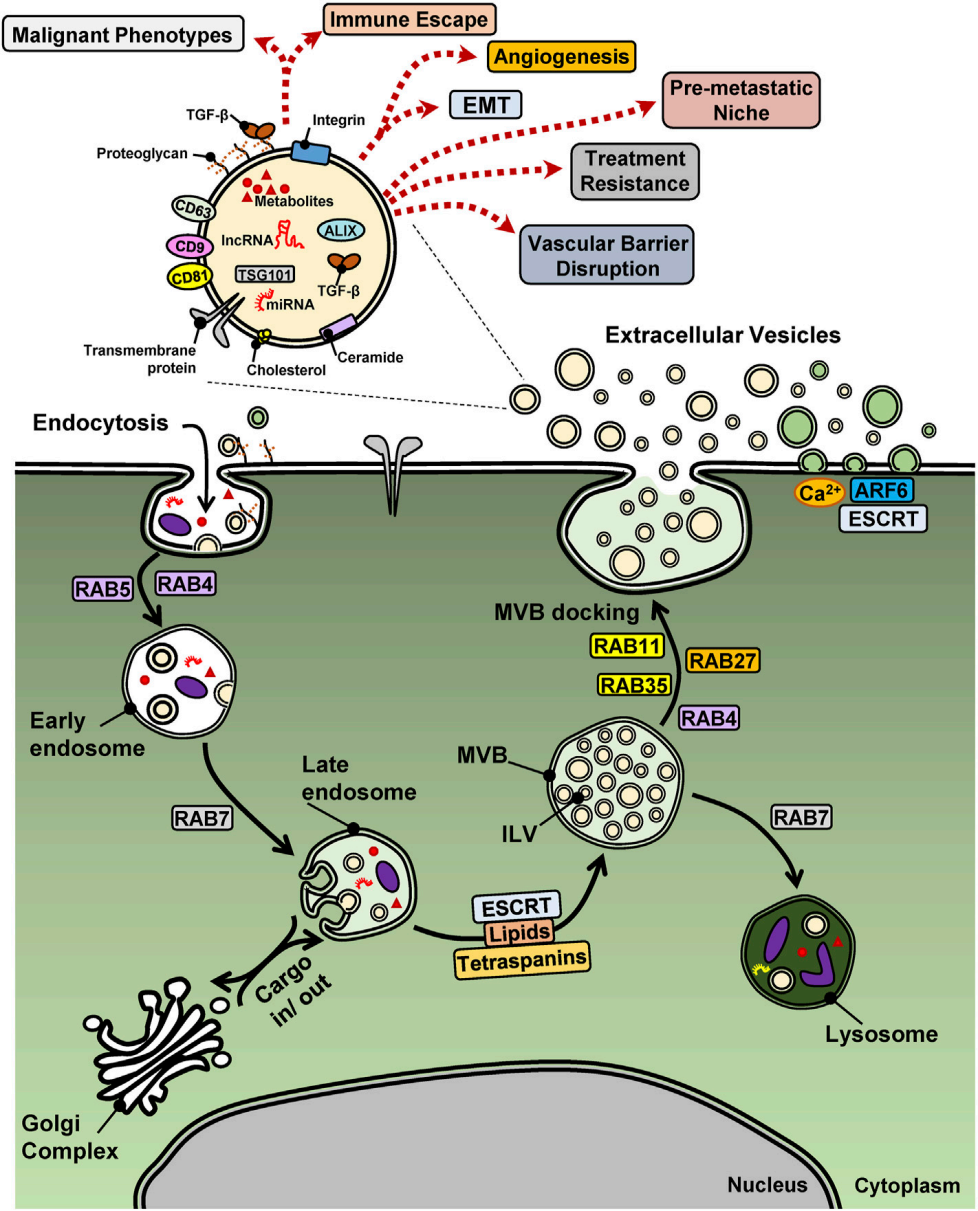
EV biogenesis and biological functions in cancer. A cancer cell is shown with surface proteins and EVs undergoing endocytosis *via* the early and late endosome and the multi-vesicular body (MVB) that shuttles protein or vesicular (intra-luminal vesicles, ILV) cargo to lysosomes or the cell surface, thus releasing exosomes (beige EVs), meanwhile outward budding of the plasma membrane releases microvesicles (green EVs). The case of apoptotic bodies generated from cells undergoing cell death is not illustrated. Key regulatory proteins of endocytosis and EV biogenesis are shown with their names boxed. A single EV is magnified in order to highlight various cargo molecules. miRNAs and lncRNAs may be viewed with potential caution as to their functional importance as EV cargo. Dotted arrows indicate diverse cell biological functions of EVs that relate to the hallmarks of cancer.

In the context of cancer, EVs are considered as important vehicles that assist the intercellular communication and the development of the microenvironment where tumors develop (Figure 1) (Liu et al., 2021; Schubert and Boutros, 2021). For example, EVs can mediate and maintain molecular gradients that lead to differential responses of the various cell types that populate the tumor microenvironment (TME), such as mesenchymal stem cells (MSCs), fibroblasts, adipocytes, neurons, immune cells and blood or lymphatic endothelial cells and pericytes (Oudin and Weaver, 2016). The preparation of metastatic sites, also known as “niches” by a process often called “seeding,” has also been linked to tumor-derived EVs that generate a proper tissue microenvironment that fosters metastatic colonization (Peinado et al., 2017). Both at the primary tumor and at metastatic sites, EVs can mediate metabolic adaptations of the tumor cells and the cells of the TME, possibly assisting tumor cell survival during the interactions of these tumor cells with multiple other cell types along the metastatic trajectory (Li and Simon, 2020; Bergers and Fendt, 2021). Special attention has recently been given to the communication mediated by EVs between tumor and immune cells, as this provides opportunities for the improvement of immunotherapy against cancer (Garner and de Visser, 2020). Whether EV-mediated processes may improve future treatment of cancer patients, remains to be evaluated (Witwer et al., 2019), yet multiple recent reports raise the utility of EVs and their molecular cargo as biomarkers with predictive and diagnostic potential that can improve cancer treatment by characterization and subclassification of tumors (Hoshino et al., 2020).

### TGF-β Signaling in Cancer

**FIGURE 2:**
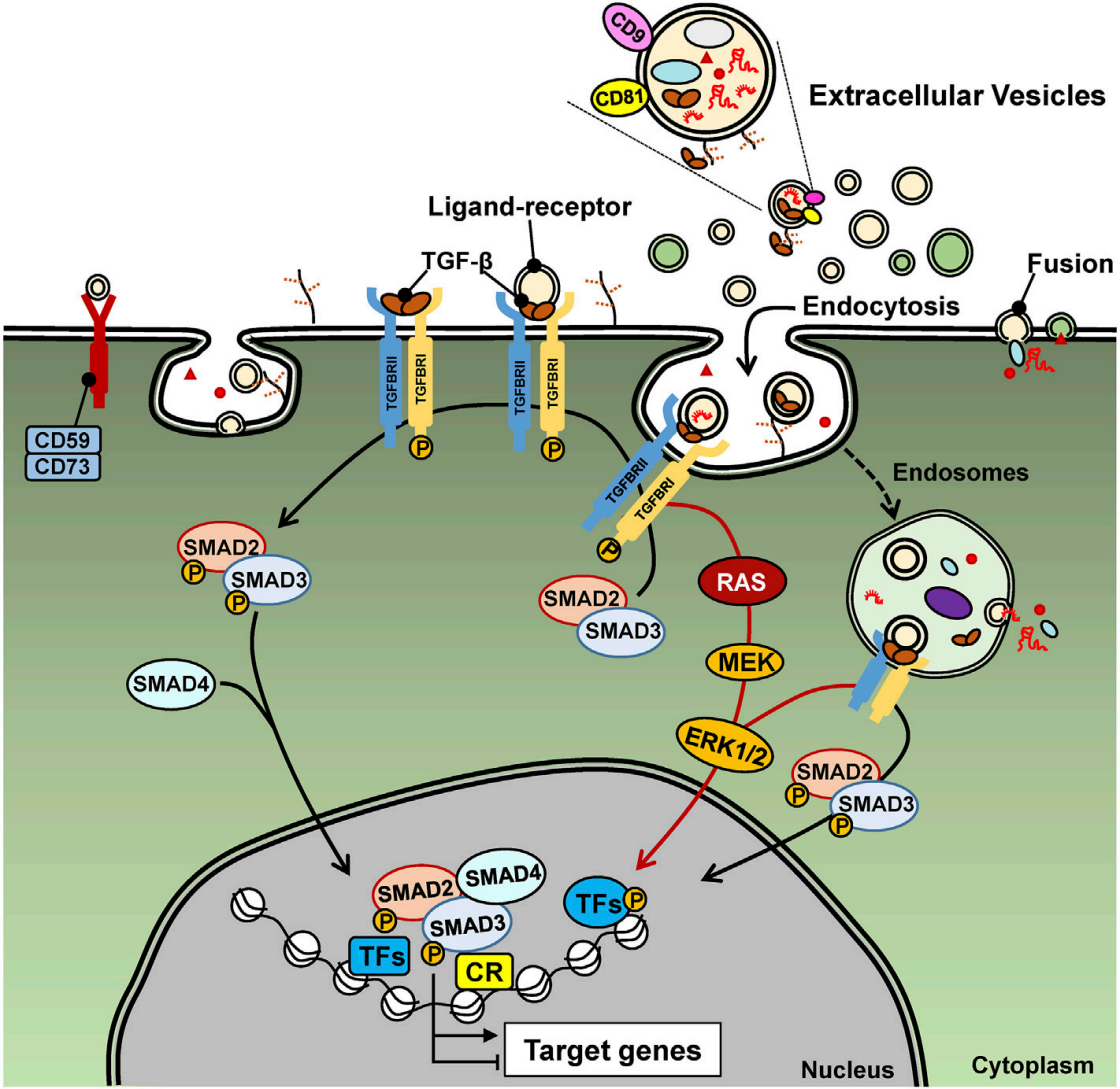
TGF-β signaling. Extracellular TGF-β (usually deposited in the ECM) and here shown as free mature TGF-β, binds to the type II and type I receptors on the cell surface, which signal *via* inter-receptor trans-phosphorylation. The type I receptor phosphorylates SMAD2 and SMAD3 that results in their oligomerization with SMAD4. The ligand-bound receptors also activate RAS, MEK, ERK and other (not shown) protein kinase signaling pathways. EV-associated TGF-β signals in the same manner, yet the ligand is presented from the surface of EVs, as endocytosis of these EVs is in progress. The signaling proteins, SMADs and MAPKs regulate gene transcription *via* direct binding to DNA (SMADs) and *via* phosphorylation of transcription factors (TF) and association with chromatin regulatory protein (CR). MiRNAs and lncRNAs are illustrated as EV cargo and may be viewed with potential caution as to the ability of EVs to deliver functional RNAs to the recipient cells that can affect TGF-β signaling either in a positive or negative manner.

Appreciating the potential of EVs in cancer biology, prompts the identification of specific molecular cargo carried in the lumen, the membrane or even bound to the surface of EVs. Among such cargo, the transforming growth factor β (TGF-β) has been steadily featured in cancer EV biology (Schubert and Boutros, 2021; Tan et al., 2021; Webber et al., 2010). This may not come with any surprise as TGF-β and other members of the TGF-β family exert versatile intercellular communication among all cell types and across metazoan evolution (Moustakas and Heldin, 2009; Tzavlaki and Moustakas, 2020). In a nutshell, the TGF-β family pathways are initiated by binding of ligands to type I and type II cell surface receptors (e.g. TGFβRI and TGFβRII in the case of TGF-β), causing their oligomerization and inducing activation of the protein kinase activity of the type I receptor (Heldin and Moustakas, 2016; Tzavlaki and Moustakas, 2020). Co-receptors also facilitate the recruitment of ligands to the signaling receptors (Heldin and Moustakas, 2016). The best studied co-receptor for TGF-β is the type III receptor (TGFβRIII, also known as betaglycan) (Heldin and Moustakas, 2016). TGFβRIII is a transmembrane proteoglycan that binds all three TGF-β ligands with high affinity and presents them to the TGFβRII and TGFβRI signaling complex. The activated TGFβRII and TGFβRI recruit and induce many signaling proteins such as protein and lipid kinases, scaffolding proteins and small GTPases, whereas some of these proteins become directly phosphorylated by the TGFβ receptors. A well understood substrate of the TGFβRI is the family of SMAD proteins (e.g. SMAD2 and SMAD3 in the case of TGF-β), which, upon phosphorylation, oligomerize with SMAD4, accumulate in the nucleus and regulate gene transcription by binding to regulatory sequences in the genome together with other transcription factors and chromatin proteins (Figure 2) (Morikawa et al., 2013; Tzavlaki and Moustakas, 2020). Several of these proteins become post-translationally modified by the action of the signaling proteins initially activated by the TGF-β receptors (Tzavlaki and Moustakas, 2020). In addition to signaling protein mediators, the TGF-β family pathways are regulated by non-protein coding RNAs, including micro-RNAs (miRNAs) and long non-coding RNAs (lncRNAs) (Janakiraman et al., 2018; Lai et al., 2020; Papoutsoglou and Moustakas, 2020).

TGF-β was isolated as a tumor-derived growth factor with potential to induce oncogenic transformation in cell models in culture and has been proven to suppress benign and pre-malignant tissue hyperplasia, but also to facilitate the development of aggressive and metastasis-prone tumors (Drabsch and ten Dijke, 2012; Derynck et al., 2021). The effects of TGF-β signaling in cancer are multiple, complex and depend on the context of the intercellular interactions, making TGF-β pathways linked to every hallmark of cancer (Drabsch and ten Dijke, 2012; Derynck et al., 2021). Prominent and extensively studied functions of TGF-β in pre-malignant tissues include the arrest of the cell cycle at the G1 phase, which is cell type-independent, and the induction of apoptosis in specific cell types, e.g. liver or prostate epithelial cells (David and Massagué, 2018). In contrast, in tumors, TGF-β is known to induce epithelial-mesenchymal transition (EMT) that fosters cancer cell invasiveness and initiation of metastasis. Moreover, TGF-β mediates cancer-associated fibroblast (CAF) to myofibroblast differentiation and tissue fibrosis, and acts as a potent suppressor of anti-cancer immunity (Webber et al., 2010; Drabsch and ten Dijke, 2012; Moustakas and Heldin, 2012; Caja et al., 2018; Derynck et al., 2021). In the following sections, these important actions of TGF-β in cancer will be discussed from the point of view of the role of EVs in each of the specific processes that mediate cancer development.

### Extracellular Vesicles and Their Βiοgenesis

One of the first reports about the relevance of EVs in tumor biology showed that tumor cells could secrete microparticles with pro-coagulant function (Dvorak et al., 1983). These particles were later renamed as microvesicles and their origins were linked to responses to stimuli that act on the cell membrane and result in vesicular shedding into the extracellular space (Hugel et al., 2005; Piccin et al., 2007). Other origins of EVs were linked to processes that regulate early endosome maturation into late endosomal compartments known as multi-vesicular bodies (MVBs). The term MVBs indicates that such vesicles can accumulate intraluminal vesicles (ILVs) in their lumen, due to a reverse budding mechanism of their membrane (Stoorvogel et al., 1991). These ILVs can later be released into the extracellular milieu as EVs/exosomes, through fusion of MVBs with the plasma membrane (Figure 1) (Johnstone et al., 1987).

Although the biogenesis of exosomes and microvesicles are different, both share membrane-trafficking processes that mediate membrane budding followed by a fission process, which generates EVs secreted inside the lumen of MVBs or in the extracellular milieu (van Niel et al., 2018). Thus, microvesicles are generated by the outward budding of the plasma membrane, a process incorporating changes in lipid bilayer composition, protein intercalation and Ca^2+^ levels, and regulated by the small GTPase ADP-ribosylation factor 6 (ARF6), which leads to the depolymerization of the actin cytoskeleton, and by TSG101 and ALIX, components of the endosomal sorting complex required for transport (ESCRT) (Figure 1) (Colombo et al., 2014). The ESCRT machinery, which comprises approximately 30 proteins that assemble into four complexes (ESCRT-0, -I, -II and -III) with associated proteins (VPS4, VTA1, ALIX), is a major regulator of EV biogenesis, driving the sorting of cargo, the membrane shape and fission of ILVs present in MVBs (Hanson and Cashikar, 2012; Hurley, 2008). Furthermore, ESCRT-independent mechanisms involve specific lipids (cholesterol, ceramide and phosphatidic acid) and transmembrane proteins, the tetraspanins (CD9, CD63, CD81 and CD82), regulating the sorting of cargo and of membranous microdomains that will be incorporated into budding EVs (van Niel et al., 2018). Members of the Ras-related protein in brain (RAB) family also regulate vesicular trafficking along the endocytic pathway, either driving the MVB to lysosome route, or the MVB to plasma membrane fusion and secretion route (Figure 1) (Stenmark, 2009; Colombo et al., 2014). Additionally, apoptotic cells also release vesicles, denominated apoptotic bodies and formed by blebbing of the plasma membrane that surrounds among other constituents, nuclear fragments. Hence, the different processes that drive EV biogenesis and the physiological state of the cell (surviving or undergoing apoptosis) lead to heterogeneous EV subpopulations, with exosomes, microvesicles or apoptotic bodies coexisting simultaneously in a given cell or tissue microenvironment.

### Extracellular Vesicles and Their Cargo

Several cargo molecules carried by EVs (proteins, nucleic acids, lipids and metabolites) (Table 1) seem to represent regulators of EV biogenesis. Moreover, the physiological cell state and extracellular stimuli modulate the molecular mechanisms inducing EV biogenesis, generating the context- and cell type-dependent shedding of EVs (van Niel et al., 2018). Among nucleic acids, non-protein coding RNAs (miRNAs and lncRNAs) have been reported to make up EV cargo (Valadi et al., 2007). However, it should be noted that RNAs in exosomes are usually in the range of 200–400 nt long, representing precursors to miRNAs or fragmented rRNAs, mRNAs and lncRNAs (Chen et al., 2010; Enderle et al., 2015; Jenjaroenpun et al., 2013; Kesimer et al., 2009). Quantitative analysis has also estimated that, on average, far less than one molecule of a given miRNA could be identified in the cargo of a single exosome (Chevillet et al., 2014). Furthermore, and in contrast to an extensive number of studies showing that EVs deliver functional RNAs to recipient cells (Table 1), it was recently reported that miRNAs are minor constituents of EVs that are rarely delivered to target cells (Albanese et al., 2021). Yet, another recent study points to a specific molecular mechanism, *via* which RNA sequences within the central body of the precursors of miRNAs are recognized by RNA-binding proteins and thus specify miRNA retention or enrichment within EVs or within cells (Garcia-Martin et al., 2022). In agreement with a more prominent role of the protein cargo carried by EVs, it was demonstrated that the main cargo attribute that drives MSC-derived exosome function is likely to be made of proteins and not RNAs (Toh et al., 2018). In summary, publications reporting the function of bioactive molecules, including proteins or RNAs, carried by tumor-derived EVs, are often based on gain or loss of function of a given cargo molecule; it thus remains possible that such genetic perturbations performed on the secreting cells, may induce changes in the amount of secreted EVs and/or in the overall molecular content of the EVs, leading to indirect effects of the perturbed molecule and not true effects of the analyzed cargo (Théry et al., 2018). Hence, ISEV has raised concern about the validation of such functional studies of EVs carrying bioactive molecules, requiring a complete characterization of the “engineered EVs,” considering at least a small-scale analysis of EV amount or common EV-associated proteins in comparison to the unperturbed EVs (Théry et al., 2018). In view of this critical evaluation of the EV cargo, all cases presented in this article (Table 1), should be considered with the appropriate caution.

**TABLE 1:** EV cargo molecules.

EV cargo				Molecular function	Process in cancer development	Recipient cell	Cancer type	Ref
Protein, metabolite, lipid	miRNA	lncRNA	EV source					
TGF-β1, TNFα, IL-6, MMPs	—	—	—	—	Tumorigenesis; Anchorage-independent growth	—	—	Redzic et al. (2013); Urciuoli et al. (2018)
Fibronectin	—	—	—	—	Tumorigenesis; Anchorage-independent growth	—	—	Antonyak et al. (2011)
—	*miR-200b*	—	—	*p27* mRNA downregulation	Tumorigenesis; Proliferation	—	CRC	Zhang et al. (2018)
—	*miR-19b; miR-92a*	—	—	*TGFBR1*, *TGFBR2* mRNA downregulation	Tumorigenesis; Proliferation	—	LUAD	Borzi et al. (2019)
—	*miR-142-3p*	—	—	*TGFBR1* mRNA downregulation	Tumorigenesis; Proliferation	—	ORCA	Dickman et al. (2017)
β-Catenin	*miR-23a*	—	—	—	EMT	—	A549, LUAD	Kim et al. (2016)
—	—	*HULC*	—	ZEB1 expression	EMT; Circulating cancer cells	—	PDAC	Takahashi et al. (2020)
—	*miR-21, miR-31, miR-145*	—	—	AR (androgen receptor)	EMT; Mesenchymal cells	—	PC	El-Sayed et al. (2017)
SLUG, SOX2	—	*MALAT1, linc-ROR*	—	—	EMT; CSCs	—	Thyroid cancer	Hardin et al. (2018)
—	*miR-424*	—	BMSC EVs	*TGFBR3* mRNA downregulation	Invasion; Metastasis	—	CRC	Zhang et al. (2021)
—	*miR-378b*	—	—	*TGFBR3* mRNA downregulation	Cancer aggressiveness; Angiogenesis	—	HCC	Chen et al. (2021a)
TGF-β	—	—	Breast milk EVs	—	EMT	Normal epithelial cell	—	Qin et al. (2016)
TGF-β	—	—	MSC EVs	—	EMT	Normal epithelial cell	—	Zhao et al. (2018)
TGF-β1	—	—	Mast cell EVs	—	EMT	—	Human LUAD cells	Yin et al. (2020)
Fibronectin	—	—	EVs	—	Migration; Metastasis	—	—	Sung et al. (2015); Sung et al. (2020)
MMPs	—	—	—	Invadopodia	Invasion	—	—	Clancy et al. (2015); Hoshino et al. (2015)
Integrin α_v_β_6_	—	—	—	LAP-TGF-β1	Migration; Metastasis	—	PC	Fedele et al. (2015)
—	—	*lncMMP2-2*	—	MMP-2	Invasion; intravasation	—	LUAD	Wu et al. (2018)
ATP	—	—	—	P2X7 receptor activates JNK, ROCK1	Migration	—	LUAD	Takai et al. (2012)
—	—	*lncRNA-ATB*	—	*miR-204-3p* sponge and TGF-β upregulation	Migration; Invasion	—	Glioma	Bian et al. (2019)
TGF-β, IL-6, TNFα, MMPs	—	—	—	Hypoxia	Pre-metastatic niche	—	PC	Ramteke et al. (2015)
Chemokines	—	—	—	Integrins	Pre-metastatic niche (endothelium)	T effector and memory cells	—	Shulman et al. (2011)
TGF-β	—	—	—	IL-6 secretion	Pre-metastatic niche	MSCs	OS	Baglio et al. (2017)
—	—	*circPACRGL*	—	*miR-142-3p*, *miR-506-3p* inhibition and TGF-β upregulation	Pre-metastatic niche (N1-N2 neutrophils)	—	CRC	Shang et al. (2020)
MET	—	—	—	BMDCs, vasculogenesis	Pre-metastatic niche	—	—	Peinado et al. (2017)
—	*miR-21-5*	—	—	SMAD7, TGF-β signaling activation	Invasion; Angiogenesis	—	Gastric cancer	Li et al. (2018)
—	—	—	—	TGF-β upregulation	Pre-metastatic niche (liver)	Kupffer cells	PDAC	Costa-Silva et al. (2015)
—	*miR-92*	—	BMDC EVs	SMAD7, TGF-β signaling activation	Pre-metastatic niche (liver)	HSCs	Liver metastasis	Hsu et al. (2020)
Integrin α_v_β_5_	—	—	—	—	Pre-metastatic niche	Kupffer cells	Liver metastasis	Hoshino et al. (2015)
Integrin α_6_β_4_, α_6_β_1_	—	—	—	—	Pre-metastatic niche	—	Lung metastasis	Hoshino et al. (2015)
ITGBL1	—	—	—	TGF-β,IL-6, IL-8	Liver metastasis	HSCs	CRC	Ji et al. (2020)
Integrin α_6_β_4_, α_6_β_1_ or α_v_β_5_	—	—	—	—	Pre-metastatic niche; Biomarkers	—	CRC to lung or liver metastasis	Ji et al. (2020)
CEMIP	—	—	—	—	Pre-metastatic niche	—	Brain metastasis	Rodrigues et al. (2019)
TGF-β, VEGF	—	—	—	—	CAFs	ADSCs	BRCA, OVCA	Cho et al. (2011); Cho et al. (2012); Song et al. (2017)
TGF-β	—	—	—	—	CAFs	MSCs	Gastric cancer	Gu et al. (2012)
—	*miR-21*	—	—	—	CAFs	HSCs	HCC	Zhou et al. (2018b)
—	*miR-10b*	—	—	PI3K downregulation	CAFs	Fibroblasts	CRC	Dai et al. (2018)
TGF-β	—	—	—	—	CAFs	Fibroblasts	Bladder cancer	Ringuette Goulet et al. (2018)
TGF-β1	—	—	TAM EVs	—	CAFs	Fibroblasts	—	Umakoshi et al. (2019)
TGF-β, TGFβRIII	—	—	—	—	Myofibroblasts	Fibroblasts	BRCA, PC	Webber et al. (2010); Webber et al. (2015)
—	*miR-17-5p*	—	—	*RUNX3* mRNA downregulation, MYC upregulation, TGF-β1 upregulation	CAFs	—	CRC	Zhang et al. (2020)
—	*miR-192, miR-215*	—	—	*Caveolin-1* mRNA downregulation, TGF-β signaling activation	CAFs	—	HNSCC	Zhu et al. (2021)
Tumor antigenic peptides	—	—	—	MHC receptor activation	Immune escape	—	Diverse tumors	Robbins and Morelli, (2014); Seo et al. (2018); Whiteside, (2016)
TGF-β1	—	—	—	MDSC accumulation	Immune escape	—	Murine BRCA	Xiang et al. (2009)
TGF-β1	—	—	—	—	Immune escape	Antigen-presenting cells	Melanoma	Düchler et al. (2019)
TGF-β1	—	—	—	—	Immune escape	T_reg_	CRC	Yamada et al. (2016)
TGF-β1, IL-12	—	—	—	—	Immune escape	—	CRC	Rossowska et al. (2019)
TGF-β1	—	—	—	—	Immune escape	NK cells	AML	Szczepanski et al. (2011)
TGF-β1	—	—	—	—	Immune escape	NK cells	CML (K562), Lung LCC	Berchem et al. (2016)
TGF-β1	—	—	—	—	Immune escape	DC, CD4^+^ T, NK	Leukemia	Huang et al. (2017)
TGF-β1, PS	—	—	—	—	Immune escape	CD8^+^ CTLs	EG7 lymphoma	Xie et al. (2009)
TGF-β1, PS	—	—	—	—	Immune escape	Macrophages	B16F10 murine melanoma	Lima et al. (2009)
Fibronectin, IL-6, MMP-10, MMP-12	—	—	—	Inflammasome	Immune escape	—	HNSCC	Bottino et al. (2021)
TIM-3?	—	—	—	—	Immune escape	M2 macrophages	MG63 OS	Cheng et al. (2021)
?	—	—	—	—	Immune escape	T_reg_	NPC	Mrizak et al. (2015)
LAMP1, MMP-9	—	—	—	—	Immune escape	B cells	ECA	Li et al. (2015)
PD-L1, TGF-β1	—	—	—	—	Immune escape	—	—	Kang et al. (2020); Mathew et al. (2020)
—	*miR-93-5p*	—	—	*FOXA1* mRNA downregulation, TGF-β3 upregulation	CAFs; Treatment resistance	—	CRC	Chen et al. (2020)
TGF-β1, TGFβRII	—	—	—	—	Treatment resistance	Keratinocytes	ORCA	Languino et al. (2016)
TGF-β	—	*Linc-ROR*	—	—	Treatment resistance	—	HCC	Takahashi et al. (2014)
—	*miR-23-5p*	—	—	*GREM2* mRNA downregulation	Treatment resistance (taxanes)	—	PC	Shan et al. (2020)
TGF-β3	—	—	—	—	Treatment resistance (cisplatin)	—	HNSCC	Rodrigues-Junior et al. (2019)
*TGFB1*, *IL-8* mRNA	—	—	—	—	Drug response biomarkers	—	Glioma	Poggio et al. (2019)
THBS2, VCAN, TNC, FN1	—	—	—	—	Biomarkers	—	Diverse tumors	Hoshino et al. (2020)
TGF-β1	—	—	—	—	Biomarkers	—	PC	Signore et al. (2021)
—	*miR-122-5p*	—	—	—	Biomarkers	—	PDAC	Zhou et al. (2018a)

Table listing EV cargo in groups (proteins, metabolites, lipids, miRNAs, lncRNAs), EV source, function of the molecule, cancer process involvement, recipient cell, cancer type and the corresponding reference. Empty entries indicate lack of information or lack of relevance. The validity of these reported EV cargoes has been criticized in the text and caution is suggested to the careful reader of the table. EV cargoes are listed in the same order as they appear in the main text. Cancer type abbreviations: AML, acute myeloid leukemia; BRCA, breast cancer; CML, chronic myelogenous leukemia; CRC, colorectal cancer; ECA, esophageal cancer; HCC, hepatocellular carcinoma; HNSCC, head-and-neck squamous cell carcinoma; LUAD, lung adenocarcinoma; NPC, nasopharyngeal cancer; ORCA, oral cancer; OS, osteosarcoma; OVCA, ovarian cancer; PC, prostate cancer; PDAC, pancreatic adenocarcinoma.

For instance, *TGFBR2* deficiency that inactivates most if not all aspects of TGF-β signaling, affects the miRNA and protein content of EVs derived by colorectal cancer (CRC) cells, indicating that TGF-β signaling may regulate EV biogenesis or secretion (Fricke et al., 2019a; Fricke et al., 2019b). It is well known that the TGF-β pathway regulates the transcriptional and post-transcriptional expression of different genes, but to date, the impact of TGF-β signaling on the content of EV cargo is yet poorly studied. Hence, the findings by Fricke et al. raise new perspectives about how TGF-β signaling could be implicated in the cell-to-cell communication mediated by vesicles (Fricke et al., 2019a; Fricke et al., 2019b).

To understand how the different molecules carried by EVs can affect the recipient cells, it is important to mention the different interaction routes of cell-to-cell communication mediated by vesicles. These can represent interactions of ligands present on the surface of EVs (e.g. TGF-β1) with cell surface receptors, inducing the activation of intracellular signaling (Figure 2). Alternatively, EVs could fuse with the cell membrane of the recipient cells and the EV content released into the cytoplasm, affecting downstream signaling (Figure 1) (Del Conde et al., 2005; Valadi et al., 2007). Nevertheless, EV uptake also occurs *via* energy-dependent, receptor-mediated endocytosis, in which EVs remain intact during and after cell entry, until specific cargo molecules interact with molecular pathways that initiate at the endocytic or phagocytic compartments (Svensson et al., 2013). In the latter context, we here discuss how molecules carried by tumor-derived EVs can affect TGF-β signaling positively or negatively in different cell types, inducing responses in recipient tumor cells, as well as in other cells in the TME.

### Extracellular Vesicles, Tumorigenesis and Epithelial-Mesenchymal Transition

Tumor-derived EVs (TDEs) can directly alter the physiology of surrounding and distant normal cells to promote cancer growth. For instance, TDEs can influence endothelial cells by inducing neo-angiogenesis and vascular leakiness, causing fibroblast differentiation into CAFs and suppressing immune cells allowing for generation of pro-tumorigenic and pro-metastatic phenotypes that lead to cancer progression and dissemination (Liu D. et al., 2016; Nabet et al., 2017; Nazarenko et al., 2010; Webber et al., 2010), aspects that are discussed later. Interestingly, disruption of EV biogenesis by inhibition of the small GTPase RAB27a, which regulates EV secretion, hinders primary tumor growth as well as metastasis of malignant cells (Bobrie et al., 2012). Increasing evidence suggests that TDEs perform these cell phenotypic changes by horizontal transfer of functional molecules/oncoproteins and activation of downstream signaling pathways in the recipient cells. For instance, EVs derived from various cancer cell types, in addition to TGF-β, transfer tumor necrosis factor-α (TNF-α), interleukin 6 (IL-6) and matrix metalloproteinases (MMPs) to normal recipient cells, promoting their proliferation, migration and anchorage-independent growth (Table 1) (Redzic et al., 2013; Urciuoli et al., 2018). Numerous studies have also indicated the role of EVs in the generation of a microenvironment permissive to tumor growth (Liu et al., 2021; Schubert and Boutros, 2021). TDEs carry fibronectin, which, once bound to integrin receptors of normal fibroblasts, promotes their anchorage-independent growth (Antonyak et al., 2011). Similarly, prostate cancer (PC) cell-derived EVs enriched in TGF-β1, induced SMAD3 and α smooth muscle actin (αSMA) expression in normal fibroblasts, promoting their differentiation to myofibroblasts (Table 1) (Webber et al., 2010).

EVs have also been reported to carry miRNAs and lncRNAs (Table 1), resulting in altered expression of tumor-suppressing or tumor-promoting genes in the recipient cells (Wei et al., 2014; Qu et al., 2016; Zheng et al., 2016; Hsu et al., 2017; Hardin et al., 2018; Ota et al., 2018; Bian et al., 2019; Borzi et al., 2019; Bier et al., 2020). As explained in detail in the previous section, these findings should be considered with caution.

In colorectal carcinoma, TGF-β1 was found to upregulate *miR-200b* levels; transfer of *miR-200b* to recipient cells *via* EVs directly targeted the 3′- UTR of the *p27* mRNA, suppressing expression of this cell cycle inhibitor, and leading to increased cancer cell proliferation (Zhang et al., 2018). In addition, EVs derived from lung adenocarcinoma cell lines increased proliferation of pre-neoplastic bronchial epithelial cells, favoring tumor growth, due to the transfer of *miR-19b* and *miR-92a*, which attenuated the expression of TGFBRI and TGFBRII in the recipient epithelial cells, where the anti-proliferative role of TGF-β is well established (Borzi et al., 2019). Furthermore, tumor-suppressive miRNAs can be selectively packaged into EVs, thus eliminating their anti-tumor function from the donor cancer cell. To this end, selective elimination of the *miR-142-3p via* EVs led to enhanced proliferation of the donor oral cancer cells as well as to induced pro-angiogenic activity in the recipient stromal cells, *via* altered expression of TGFBRI (Dickman et al., 2017).

An integral process involved in cancer progression, first proposed more than a 100 years ago by Santiago Ramon y Cajal, is the EMT (Nieto et al., 2016). EMT is linked to the modification of the primary TME in order to facilitate invasion. This is achieved by reassembly of epithelial cell-cell adhesions, modification of cell-extracellular matrix (ECM) interactions, reorganization of cytoskeleton and remodelling of the secreted extracellular proteins (Derynck and Weinberg, 2019; Moustakas and Heldin, 2012; Nieto et al., 2016). Currently, the EMT status in a primary tumor is validated by the expression of a combination of epithelial and mesenchymal genes, as was established in a breast cancer (BRCA) microarray (Sarrio et al., 2008). As already introduced, the TGF-β pathway prominently induces EMT (Moustakas and Heldin, 2012). TGF-β signaling is directly coupled to the transcriptional induction of a cohort of transcription factors that initiate the EMT (EMT-TFs), such as SNAI1, SNAI2, ZEB1, ZEB2, TWIST1 and TWIST2, many of which also receive signals from TGF-β that control their activity (de Herreros et al., 2010; Moustakas and Heldin, 2012; Nieto et al., 2016). Induction of EMT in A549 human lung adenocarcinoma cells upon TGF-β1 treatment altered the protein and miRNA cargo of EVs, which reflected the phenotypic condition of the cells they derived from (Kim et al., 2016). Although the EVs were not properly characterized in this study, the data showed an increment of β-catenin and *miR-23a* levels in A549-derived EVs treated with TGF-β1 (Kim et al., 2016). In the same cell model, β-catenin mediates signaling that promotes EMT (Tian and Phillips, 2002), whereas *miR-23a* downregulates the adherens junction protein E-cadherin (Cao et al., 2012). Moreover, stimulation of human pancreatic ductal adenocarcinoma cells by TGF-β induced the lncRNA *HULC*, which caused ZEB1 upregulation and promoted EMT (Takahashi et al., 2020). *HULC*-containing EVs caused increased *HULC* levels in recipient human pancreatic ductal adenocarcinoma cells (Table 1), which resulted in further induction of the EMT program (Takahashi et al., 2020). In addition, *HULC* encapsulated in EVs was upregulated in the serum of pancreatic ductal adenocarcinoma patients, suggesting that the EVs and their *HULC* cargo might serve as diagnostic biomarkers (Takahashi et al., 2020).

Mesenchymal cell TDEs can transform normal or pre-malignant epithelial cells in experimental systems. For instance, EVs derived by mesenchymal PC cells triggered phenotypic changes in the recipient androgen-dependent epithelial PC cells by direct inhibition of androgen receptor signaling and activation of TGF-β signaling (El-Sayed et al., 2017). The miRNAs *miR-21*, *miR-31* and *miR-145* directly regulate androgen receptor levels and appeared elevated in both mesenchymal-derived TDEs and in recipient carcinoma cells (Table 1), suggesting a horizontal transfer of cargo, which promoted the survival of a more plastic (EMT) state and generation of an aggressive PC cell subpopulation (El-Sayed et al., 2017). In addition, EVs isolated from cancer stem cells upon culture with normal thyroid cells upregulated the lncRNA *MALAT1* and the *long intergenic non-protein coding RNA, regulator of reprogramming* (*linc-ROR*) levels (Table 1), as well as the EMT transcription factor SNAI2 and the stem cell transcription factor SOX2 (Hardin et al., 2018). The cells receiving these EVs presented increased proliferative and invasive ability when compared to control cells (Hardin et al., 2018).

Disruptions of TGF-β signaling in CRC drives tumor progression (Itatani et al., 2019). In this context, EVs derived from bone marrow-derived MSCs (BMSCs) carrying *miR-424* induced an aggressive phenotype in CRC cells *in vivo* by targeting *TGFBR3* (TGFβRIII) transcripts (Zhang et al., 2021). Similarly, downregulation of *TGFBR3* expression in hepatocellular carcinoma (HCC) was driven by HCC cell-derived EVs carrying *miR-378b*, which increased progression and angiogenesis (Chen W. et al., 2021). In these two examples, downregulation of TGFβRIII is thought to inhibit physiological TGF-β signaling, which acts homeostatically, and thus, the loss of the co-receptor TGFβRIII might be equivalent to the loss-of-function mutations in TGFβRII that sometimes are required for the progression of CRC. Remarkably, in addition to malignant cells, EVs derived from normal cells contain high TGF-β levels, i.e. EVs secreted by breast epithelial cells of healthy lactating women in their milk and EVs secreted from human umbilical cord MSCs, were reported to induce EMT and malignant transformation of both cancer and benign epithelial cells (Table 1), as observed by activation of the canonical and non-canonical TGF-β signaling pathways, and altered expression or assembly of EMT related proteins (E-cadherin, vimentin, αSMA, filamentous actin) in the recipient cells (Qin et al., 2016; Zhao et al., 2018). Furthermore, mast cell-derived EVs rich in TGF-β1 on their surface, induced EMT when taken up by epithelial human lung adenocarcinoma cells as observed by increased mRNA and protein levels of EMT-TFs (TWIST1, SNAI1, SNAI2) and of induced phosphorylation of cellular proteins involved in EMT (TGM2, annexin-A1, VACM1, Chrombox3), cell-cell junctions (E-cadherin, N-cadherin), cell-ECM interactions (MMP-2, MMP-9) and cell proliferation (c-KIT) (Yin et al., 2020). Altogether these findings suggest that key regulators of TGF-β signaling can be shed extracellularly as content of EVs and thus, they can induce signaling that modulates the adjacent and distant TME in order to culminate tumor progression and metastasis initiation.

### Extracellular Vesicles and TGF-β as Mediators of Cancer Metastasis

Metastatic outgrowth of the primary tumor to distant organs is the major cause of death due to cancer. This requires the invasion of neoplastic cells from the primary tumor through the basement membrane and dissemination *via* the circulation. Accumulating evidence suggests that TDEs may promote metastases at secondary sites, and that the EV-mediated pro-metastatic signal transmission can take place either within the primary tumor or at distant organs and/or tissues contributing to premetastatic niche formation.

In the primary tumor, EVs show a prominent role in modifying the motility of cancer cells and their invasive abilities. Secretion of EVs is required for *in vivo* movement and cell migration of cancer cells by promotion and stabilization of leading-edge adhesive protrusions (Sung et al., 2015). Development of a pH-sensitive EV reporter (pHluo_M153R-CD63) that allowed the monitoring of cellular interactions with EVs, demonstrated that the EVs are secreted at the front edge of the migrating cells and can be used by other cell types as migrating tracks in 2D and 3D tissue culture environments (Sung et al., 2020). These cells also leave behind exosome trails and cancer cells migrating towards the leading cells in the migratory trail can actively endocytose the layered EVs, which fuel the transmission of migratory behavior (Sung et al., 2020).

EVs also act as carriers of ECM components promoting adhesion and altered cell-ECM interactions. For example, fibronectin is sorted into the cargo of EVs after integrin receptor binding and promotes cell motility (Sung et al., 2015). Furthermore, it has been shown that EVs contribute to the formation of invadopodia or acquisition of an amoeboid mode of migration *via* MVB-dependent transfer of MMPs (Hoshino et al., 2013; Clancy et al., 2015). Another study demonstrated that PC cell-secreted EVs that carry high levels of integrin α_v_β_6,_ after delivery to α_v_β_6_-negative PC cells allowed binding of the inactive latency-associated peptide (LAP)-TGF-β form, promoting its activation and the release of mature TGF-β, whose signaling induces alterations of the ECM and mediates cell migration (Fedele et al., 2015). In the same line, TGF-β-treated human lung adenocarcinoma cells secrete EVs that are enriched in *lnc-MMP2-2*, which promotes the expression of MMP-2, regulating migration and invasion of lung cancer cells and intravasation into the vasculature (Wu et al., 2018). Alternatively, TGF-β1 stimulation of human lung adenocarcinoma cells can promote exocytosis of ATP *via* vesicles, which in turn activates the ionotropic P2X7 receptor that promotes actin remodeling through activation of the Jun N-terminal kinase or Rho kinase leading to increased lung cancer cell migration (Takai et al., 2012).

By sequestering certain miRNAs, specific lncRNAs are involved in the epigenetic regulation of gene expression in several diseases including cancer (Su et al., 2021). As an example, TGF-β signaling downregulates *miR-622*, which normally targets the lncRNA *HULC* and attenuates cell invasion and migration by suppression of EMT signaling *via* EV transfer (Takahashi et al., 2020). Moreover, glioma cell-derived exosomal *lncRNA activated by TGF-*β (*lncRNA-ATB*) suppresses *miR-204-3p* in an argonaute 2-dependent manner in recipient astrocytes, causing activation of migration and invasion of glioma cells by induction of TGF-β signaling (Bian et al., 2019).

Development of hypoxia is a common pathophysiological condition observed during tumor growth and is characterized by limited supply of oxygen and nutrients to the cells of the core of the tumor mass. This condition induces angiogenesis and activates altered metabolic pathways leading to increased migration of the invasive tumor front. Thus, EVs derived from hypoxic PC cells contain elevated numbers of proteins implicated in EMT and pre-metastatic niche formation (TGF-β2, IL-6, TNF1α, MMPs, Table 1) (Ramteke et al., 2015). The cargo proteins in these EVs regulate adherens junctions, by downregulation of E-cadherin and accumulation of nuclear β-catenin, and by remodeling of the actin cytoskeleton, thus enhancing the motility and invasiveness of the PC cells (Ramteke et al., 2015). In another case, hypoxia enhanced TGF-β signaling in cancer cells, which promoted alternative splicing of hMENA, a cytoskeletal remodeller during EMT that supports fast actin polymerization, promoted cell migration and invasiveness (Ahuja et al., 2020).

### Extracellular Vesicles and the Metastatic Niche

The contribution of EVs in malignant progression by aiding in the formation of premetastatic niches can be related to the increased number of tumor-derived EVs present in the blood circulation of cancer patients (Logozzi et al., 2009; Baran et al., 2010; Galindo-Hernandez et al., 2013; Baglio et al., 2017) and to the fact that elevated levels of several EV cargoes have been associated with poor prognosis of cancer patients with metastatic progression (Peinado et al., 2012). Interestingly, injections of metastatic cell-derived EVs into the mouse blood circulation, induced formation of a pre-metastatic niche even in the complete absence of tumor cells (Grange et al., 2011; Peinado et al., 2012; Costa-Silva et al., 2015; Hoshino et al., 2015; Liu Y. et al., 2016). After generation of the niche, incoming tumor cells communicate with surrounding fibroblasts, endothelial and immune cells by receptor-mediated cell-cell interactions, *via* paracrine secretion of growth factors, chemokines and cytokines and *via* EVs. Interestingly, vascular endothelial cell-derived chemokines have been shown to be stored in vesicles which docked on actin fibers beneath the endothelial plasma membrane (Shulman et al., 2011). These chemokines were released at lymphocyte-endothelial synapses, allowing for establishment of contacts between adhesive integrins and T effector and memory cells within the inflamed endothelia (Shulman et al., 2011). Furthermore**,** using a zebrafish model, it was assessed how exogenous melanoma MemBright-labeled EVs circulate in the blood flow and how they are internalized by normal cells during formation of a pre-metastatic niche (Hyenne et al., 2019). Endothelial cells and macrophages were the major circulating cells that received EVs, and these cell types presented increased uptake efficiency of the tumor-derived EVs for degradation in lysosomes (Hyenne et al., 2019).

Plethora of studies suggest that molecular cargo released from EVs can “educate” cells to activate signaling cues favoring metastatic development mainly by induction of inflammation, immune suppression, vascular leakiness and stromal cell activation (Liu Y. et al., 2016; Nabet et al., 2017; Nazarenko et al., 2010). EV-transduced TGF-β signaling has been shown to underlie many such processes (Table 1). For example, EVs from metastatic osteosarcoma cells have been shown to carry elevated levels of a membrane-associated form of TGF-β that interacts with its receptor on the surface of MSCs and “educates” them to produce IL-6 and thus trigger a proinflammatory loop favoring metastatic seed and progression (Baglio et al., 2017). Potential mechanisms of action of such EVs may relate to a special conformation that EV-bound TGF-β takes, making this TGF-β capable of activating not only TGF-β receptors but also alternative signaling receptors that could act as co-receptors in this case. Such a mechanism remains to be experimentally confirmed. Alternatively, specific cargoes in EVs, such as inflammatory miRNAs may cooperate with TGF-β and activate Toll-like receptor signaling in the MSCs, as is the case for *miR-21* and *miR-29a* action on Toll-like receptors of immune cells during metastasis (Fabbri et al., 2012). Another plausible mechanism is that EV surface cargo signals together with TGF-β, thus providing a combinatorial message that mediates the MSC response in terms of IL-6 secretion, as is the case of fibroblast to myofibroblast differentiation by a combinatorial action of EV-carried TGF-β together with heparan sulphate, also carried on the EV surface (Webber et al., 2015). Irrespective of the specific mechanism, when injected into a preclinical mouse model, the “educated” MSCs promoted osteosarcoma growth and formation of lung metastasis, supporting the establishment of a tissue microenvironment favoring tumor growth and metastasis formation through the induction of the pro-inflammatory IL-6/STAT3 pathway (Baglio et al., 2017). Colorectal carcinomas secrete EVs carrying the non-coding RNA *circPACRGL* that regulates the expression of TGF-β by binding to *miR-142-3p* and *miR-506-3p*, promoting cancer cell proliferation and metastasis, mediated by the transformation of N1 neutrophils to N2 in the TME (Shang et al., 2020). Furthermore, the contribution of bone marrow derived cells (BMDCs) in metastasis has been well established (Kaplan et al., 2005; Hara et al., 2017; Xu et al., 2017). In this context, it was demonstrated that TDEs first recruit BMDCs through upregulation of pro-inflammatory molecules at premetastatic sites, and second, educate BMDCs to support tumor vasculogenesis, invasion and metastasis by horizontal transfer of the MET oncoprotein to bone marrow progenitors (Peinado et al., 2012). Alternatively, gastric cancer-derived exosomal *miR-21-5p* that targets SMAD7, an inhibitor of TGF-β signaling, has been shown to promote mesenchymal transition of peritoneal mesothelial cells, a process that promotes invasion through matrix remodeling and angiogenesis of the peritoneum (Li et al., 2018).

Organ-specific metastasis, the tendency of primary tumors to develop secondary malignancies in specific organs, has only recently started being understood. Several studies suggest that TDEs prepare the microenvironment at future metastatic sites and mediate non-random patterns of metastasis. For example, EVs secreted from pancreatic ductal adenocarcinoma (PDAC) promoted the formation of a pre-metastatic niche in the liver and thus increased the liver metastatic burden (Costa-Silva et al., 2015). Uptake of EVs by Kupffer cells in the liver induced the TGF-β signaling pathway *via* activation of the macrophage migration inhibitory factor (MIF), a known mediator of liver inflammation and fibrosis. This in turn promoted fibronectin production that arrested bone marrow-derived macrophages in the liver, establishing alterations of the ECM that supported metastasis (Costa-Silva et al., 2015). EVs derived from BMDCs of mouse lung tumors, contain *miR-92*, which promotes metastasis to the liver (Hsu et al., 2020). This was achieved by enhancement of TGF-β signaling in hepatic stellate cells (HSCs) (Table 1) through *1*) direct pairing of *miR-92* to the 3’ UTR of *SMAD7*, causing SMAD7 protein suppression and TGF-β pathway de-repression, *2*) accumulation of immunosuppressive cells, and *3*) upregulation of collagen type I, supporting cancer cell and myeloid-derived suppressor cell (MDSC) attachment (Hsu et al., 2020).

The targeting of EVs to specific recipient cells and internalization is proposed to depend on the presence of ECM-related proteins that promote EV adhesion to certain organs thereby initiating pre-metastatic niche formation. For example, EVs expressing the integrins α_ν_β_5_ mediated liver tropism by binding to Kupffer cells, whereas EVs expressing the integrins α_6_β_4_ and α_6_β_1_ mediated lung tropism by binding to lung fibroblasts and epithelial cells (Table 1) (Hoshino et al., 2015). Thus, integrins transported by EVs, apart from inducing adhesion to specific organs, “educate” these organs, creating an environment where metastatic cells could grow and form secondary neoplasia (Hoshino et al., 2015). In the same line, CRC cells secrete EVs carrying integrin β-like 1 (ITGBL1) to the circulation, activating fibroblasts and stellate cells in the lung and the liver, which in turn secrete growth factors and cytokines, including TGF-β, IL-6 and IL-8 that promote metastatic cancer growth and invasiveness (Ji et al., 2020). Interestingly, the content of circulating TDEs secreted from CRC cells predicted the metastatic site; integrins α6, β1 and β4 were high in plasma EVs of colorectal patients showing metastasis to the lung, while high EV integrin α5 and β5 correlated with liver metastasis of different colorectal patients (Ji et al., 2020). It is likely that there are more mechanisms through which EVs promote organ-specific metastasis. Bone-tropic EVs were reported to induce vascular leakiness in the lung instead of expressing a specific repertoire of integrins (Hoshino et al., 2015). In addition, cell migration-inducing and hyaluronan-binding protein (CEMIP) levels were elevated in EVs from metastatic cells of the brain but not in EVs from lung or bone metastasis (Rodrigues et al., 2019). CEMIP triggered vasculogenesis and promoted a pro-inflammatory state in the brain which supported metastatic colonization (Rodrigues et al., 2019). Altogether these findings provide examples on how EVs can “educate” a plethora of cell types in order to generate a metastatic microenvironment permissive of successful engraftment of incoming tumor cells. Of significance is the fact that EVs, based on their cargo, display specific preference for target organs/tissues and thus support non-random patterns of metastasis.

### Extracellular Vesicles and Cancer-Associated Fibroblasts

We briefly referred to CAFs in the EMT section. CAFs are important cell types of the TME. They are derived from resident, tissue fibroblasts, from infiltrating MSCs originating from the bone marrow, occasionally from adipocytes and possibly some other sources, and reside within the TME, where they secrete ECM proteins and enzymes that remodel the ECM (Östman and Augsten, 2009; Sahai et al., 2020; Chen Y. et al., 2021). CAFs are often defined negatively, being non-epithelial, non-endothelial, non-immune and non-hematopoietic elongated cells that are non-cancerous, in other words they lack the genetic mutations that define a cancer, and consequently, CAFs cannot be confused with mesenchymal cells generated *via* EMT (Sahai et al., 2020; Chen Y. et al., 2021). TGF-β “activates” CAFs, in other words induces their differentiation to a myofibroblastic phenotype that is highly secretory and contractile. The high secretory function of CAFs is connected to the production of EVs. We will here discuss two main actions of EVs in CAF biology: the role of EVs in CAF generation and the role of EVs in myofibroblast differentiation in various tumor types.

EVs derived from ovarian or BRCA cells cultured *in vitro*, induced a myofibroblast phenotype to adipose tissue-derived MSCs (ADSCs) (Cho et al., 2011; Cho et al., 2012; Song et al., 2017). TGF-β1 carried by the EVs was a key mediator of the myofibroblast phenotype, although the EVs carried additional growth factors, such as vascular endothelial growth factor (Table 1). Upon incubation of the EVs with the ADSCs, the differentiating cells upregulated expression of their TGF-β receptors and activated TGF-β signaling, which explains why inhibition of TGF-β receptor kinase activity blocked the differentiation process (Cho et al., 2011; Cho et al., 2012; Song et al., 2017). Unexpectedly, the ovarian cancer EVs induced SMAD2 or AKT signaling in the differentiating ADSCs, depending on the cancer cell line where the EVs were isolated from, an observation that deserves further investigation and explanation (Cho et al., 2011). Similarly, EVs produced by gastric cancer cells induced CAF generation from human umbilical cord MSCs *via* activation of TGF-β/SMAD2 signaling (Gu et al., 2012). EVs produced by HCC cells acted on isolated HSCs causing generation of CAFs that secreted TGF-β1 (Zhou Y. et al., 2018). In addition to MSCs, incubation of resident vesical fibroblasts or primary fibroblasts with EVs derived either from bladder cancer cells or from CRC cells, generated CAFs (Dai et al., 2018; Ringuette Goulet et al., 2018). The CRC EVs induced secretion of TGF-β1 and expression of αSMA, a hallmark cytoskeletal protein of myofibroblasts, when incubated with primary fibroblasts, and the resulting CAFs enhanced CRC cell proliferation *in vitro* and CRC in recipient mice where the latter cells were xenografted together with the CAFs (Dai et al., 2018). On the other hand, bladder cancer EVs carried a substantial proportion of secreted TGF-β1, induced TGF-β/SMAD2 signaling in vesical fibroblasts and CAF differentiation was inhibited by a TGF-β receptor kinase inhibitor (Ringuette Goulet et al., 2018). Induction of high expression of fibronectin in response to TGF-β carried by EVs promotes invasion of fibroblasts mediated by binding of fibronectin to integrin α_5_β_1_ on their surface (Chanda et al., 2019). A final example of EV-mediated CAF generation stems from studies of tumor-associated macrophages (TAMs), which secrete EVs carrying TGF-β1, and which upon interaction with fibroblasts induced CAFs (Table 1), but also acted on peritoneal mesothelial cells that responded with EMT, and on endothelial cells, in a gastric epithelium invasive model (Umakoshi et al., 2019). This model is relevant to the generation of a pro-metastatic niche, as discussed earlier, and provides evidence for a strong ability of the EVs to penetrate the gastric parenchyma.

Observations of myofibroblast differentiation of resident fibroblasts or CAFs are also abundant. Comparative analysis of EVs secreted by salivary adenoid cystic carcinoma cells relative to EVs secreted by CAFs from the same tumor type, showed that the CAF EVs carried a stronger potential of generating the pre-metastatic niche to the lung; the EVs acted on lung fibroblasts that generated CAFs in the metastatic colony area (Kong et al., 2019). As discussed in the tumorigenesis section, EVs from breast or prostate carcinoma cells that carry TGF-β and TGFβRIII co-receptor on their surface, induced stromal fibroblast to myofibroblast differentiation with strong αSMA expression (Webber et al., 2010; Webber et al., 2015). An important finding made in these studies was that the TGF-β carried by EVs was more potent than recombinant human TGF-β1, which was provided to the same responding fibroblasts, in terms of pro-tumorigenic and pro-angiogenic activity (Webber et al., 2010; Webber et al., 2015). The generation of the EVs required intact RAB27a GTPase activity and the response of the fibroblasts to TGF-β depended on the heparan sulphate content of the EVs, possibly reflecting the function of the co-receptor function of TGFβRIII (Webber et al., 2015). A possible reason why TGF-β carried by EVs is superior to TGF-β secreted by cancer cells, is the presence of receptors in the same EVs. Thus, EVs secreted from stromal fibroblasts of oral squamous cell carcinoma carry TGF-β1 and TGFβRII (Table 1) and mediate drug resistance to recipient oral squamous cell carcinoma keratinocytes (Languino et al., 2016). Fibroblast-derived EVs transferred fully competent TGF-β responses in patient-derived keratinocytes carrying mutations in *TGF*β*RII* and thus lacking active receptor signaling (Languino et al., 2016). This mechanism whereby tumor cells mutate components of the TGF-β pathway, and surrounding stromal cells complement this defect by utilizing their EVs as a vehicle for communication, highlight the complexity of signaling events taking place during cancer development.

### Extracellular Vesicles Carry miRNAs To Regulate CAF Biology

Based on the possibility that miRNAs are cargo of EVs secreted by cancer cells, it is appropriate to discuss a few molecular mechanisms controlled by such miRNAs and which center around the TGF-β pathway, although the validity of these mechanisms should be viewed critically. In the example of HCC EVs that generate CAFs out of HSCs, the EV cargo of relevance is *miR-21* (Table 1), which directly downregulates the PTEN phosphatase, thus promoting phosphoinositide 3′ kinase (PI3K) activity and further releasing the activity of the AKT protein kinase pathway, which is required for initiation of CAF differentiation (Zhou Y. et al., 2018). Based on its mechanistic role, *miR-21* levels measured in EVs isolated from the serum of HCC patients correlated with CAF to myofibroblast differentiation and the vascularity of the tumors (Zhou Y. et al., 2018). Focusing on the same signaling pathway, the CRC EV mechanism that generates CAFs involved the cargo *miR-10b* (Table 1), whose target was the catalytic subunit of PI3K (PIK3CA) (Dai et al., 2018). This example contrasts the previous data on *miR-21*, considering that PIK3CA was downregulated by EVs carrying the *miR-10b*. Consequently, PI3K/AKT signaling activity was reduced and the recipient fibroblasts exhibited reduced proliferation, yet they turned on expression of TGF-β1 and αSMA, which mark the differentiation of CAFs (Dai et al., 2018). Thus, CAF differentiation is linked to the inhibition of progenitor fibroblast proliferation. Two more examples from CRC exemplify the complexity of the molecular networks involved in CAF function during cancer development. Pro-metastatic actions of EVs secreted by CAFs in CRC are associated with cargo *miR-17-5p* (Table 1), which downregulates the transcription factor RUNX3 (Zhang et al., 2020). RUNX3 and c-MYC form complexes on the regulatory sequences of the *TGFB1* gene and RUNX3 suppresses the transcriptional activity of c-MYC, which positively induces expression of TGF-β1, the latter promoting CRC cell invasiveness; thus, when CAFs secrete their EVs, they provide *miR-17-5p* to CRC cells, negatively regulating RUNX3 and consequently de-repressing the *TGFB1* gene *via* c-MYC. The newly synthesized TGF-β1 from CRC cells feeds back to the CAFs and further enhances CAF differentiation, enforcing higher secretion of EVs enriched in *miR-17-5p*. It is worth noting that *miR-17-5p*, in addition to RUNX3, negatively regulates many components of the TGF-β signaling pathway, yet, whether TGF-β regulates expression of *miR-17-5p* remains to be examined. CRC CAFs can also generate EVs carrying *miR-93-5p*, whose target is the *FOXA1* mRNA (Table 1), encoding a transcriptional repressor of the *TGFB3* gene (Chen et al., 2020). This mechanism has functionally been linked to escape from radiation-mediated cell death of CRC cells *in vitro* in xenografted mice (Chen et al., 2020). Thus, EVs secreted from CAFs act on CRC cells, causing de-repression and expression of TGF-β3, *via* the action of *miR-93-5p* on FOXA1; the increased amounts of TGF-β3 contributed to the proliferation after escape from apoptosis that was induced by radiation (Chen et al., 2020). However, the proposed anti-apoptotic or pro-survival mechanism of TGF-β3 in this model requires further analysis. Lastly, EVs secreted from hypoxic head and neck squamous cell carcinoma (HNSCC) cells carry TGF-β1 as cargo that promotes CAF differentiation in co-culture experiments (Table 1); the EVs also carry *miR-192* and *miR-215*, which downregulate the caveolar protein caveolin-1, an established negative regulator of TGF-β signaling (Zhu et al., 2021). In this manner, the EVs generate a positive feedback loop whereby the EV-carried TGF-β is permitted to signal on recipient cells, and thus generate more TGF-β, which enriches the HNSCC with more CAFs. These examples highlight mechanisms whereby CAFs influence carcinoma cell invasiveness but also induction of CAF differentiation by engaging carcinoma cell-derived EVs carrying specific miRNAs that directly or indirectly impact on expression and signaling activation of the TGF-β pathway. As repeatedly noted in this article, such miRNA-based mechanisms should be viewed with appropriate caution.

### Regulation of Cancer Immunity by Extracellular Vesicles

The content of EVs secreted by cancer and stromal cells in the TME have been proposed to stimulate or suppress the activity of immune cells, including their progenitor cells. In this context, EVs can directly expose antigens in their surface, which are recognized by major histocompatibility receptors, or indirectly the EVs induce antigen presentation by transferring tumor antigenic peptides to antigen-presenting cells (Robbins and Morelli, 2014; Whiteside, 2016; Seo et al., 2018). Strong evidence supports induction of local immunosuppressive responses by tumor-derived EVs, through the generation of MDSCs (comprising myeloid progenitor cells, immature macrophages, granulocytes, and dendritic cells - DCs), which drive the function of regulatory T (T_Reg_) cells, inhibiting anti-tumoral responses (Huber et al., 2005; Robbins and Morelli, 2014). Hence, since TGF-β signaling can modulate immunosuppressive activities in several innate and adaptive immune cells (Batlle and Massagué, 2019; Derynck et al., 2021), in this section we discuss how different tumor-derived EVs promote immune evasion through TGF-β signaling.

In light of this, TDEs isolated from murine mammary adenocarcinoma carried functional TGF-β (Table 1), which effectively induced the accumulation of MDSCs, a process that could be blocked by pre-incubating the TDEs with an anti-TGF-β antibody (Xiang et al., 2009). Moreover, TGF-β transported by melanoma-derived EVs contributed to the promotion of a suppressive phenotype by antigen-presenting cells (Düchler et al., 2019), while the EVs released by CRC loaded with TGF-β1 induced phenotypic alteration of T to T_Reg_-like cells through activating TGF-β/SMAD and inactivating MAPK signaling (Yamada et al., 2016). Additionally, EVs purified from MC38 colon carcinoma cells overexpressing IL-12 and deprived of TGF-β1 by transfecting shRNA molecules targeting *TGFB1*, efficiently inhibited tumor growth and induced anti-tumor immunity, together with DC-based vaccines (Rossowska et al., 2019).

In a different cancer context, EVs isolated from the sera of a small cohort of newly diagnosed acute myeloid leukemia patients carried TGF-β1 (Table 1), apart from being positive for expression of classical EV and myeloid blast markers (CD34, CD33, and CD177) (Szczepanski et al., 2011). These circulating patient-derived EVs decreased natural killer group 2D (NKG2D) receptor levels and reduced the cytotoxicity of natural killer (NK) cells. In addition, an anti-TGF-β1 antibody blocked efficiently the EV-mediated suppression of NK cell function and the concomitant NKG2D downregulation (Szczepanski et al., 2011). Similarly, EVs purified from K562 (chronic myelogenous leukemia) cells and IGR-Heu (lung large cell carcinoma) under hypoxic conditions carried TGF-β1 (Table 1) and decreased NK cell suppressive activity by reducing NKG2D expression (Berchem et al., 2016). Moreover, EVs derived from *TGFB1*-silenced leukemic cells, promoted DC activation, facilitated CD4^+^ T-cell proliferation and Th1 cytokine secretion, and further stimulated cytotoxic responses in lymphocytes and NK cells when compared to the control leukemia-EVs (Huang et al., 2017). Thus, by treating mice with leukemia-derived EVs carrying lower TGF-β1 levels prolonged animal survival, suggesting that such EVs were more effective in both protective and therapeutic antitumor tests than non-modified EVs carrying a higher load of TGF-β1 (Huang et al., 2017).

Of note, molecules constitutively present on EV surfaces such as milk fat globule EGF and factor V/VIII domain-containing gene (MFGE8), tetraspanins, and externalized phosphatidylserine (PS) can mediate the interaction of tumor-derived EVs with immune cells (Hao et al., 2007; Robbins and Morelli, 2014; Thery et al., 1999). Tolerogenic EVs isolated from EG7-lymphoblasts undergoing irradiation-induced cell death were enriched in PS and histone H3 and suppressed DC_OVA_-stimulated CD8^+^ cytotoxic T lymphocyte (CTL) responses, *via* the induction of CD8^+^ T-cell anergy and type 1 regulatory CD4^+^ T-cell responses (Xie et al., 2009). Furthermore, the irradiation-induced apoptosis led to an increase in both TGF-β1 levels on cells and in their secreted EVs due to the activation of nuclear factor of activated T-cells (NF-AT), a transcription factor positively regulating the *TGFB1* promoter (Xie et al., 2009). Thus, an anti-TGF-β1 antibody was able to block the EV-mediated immune suppression through CD8^+^ CTL responses and anti-tumor immunity *in vivo* (Xie et al., 2009). Furthermore, PS presented on EVs isolated from B16F10 malignant murine melanoma cells, elicited anti-inflammatory responses on macrophages by inducing TGF-β1 secretion and enhancing the metastatic potential of B16F10 cells in C57BL/6 mice, while these effects were abrogated when the PS on EVs was blocked with annexin V (Lima et al., 2009). In addition to inflammatory responses mediated by macrophages, the inhibition of inflammasomes is another mechanism used by tumor cells to escape from the immune system (Ghiringhelli et al., 2009). Based on this, the NLRP3 (nucleotide-binding oligomerization domain (NOD)-, leucine-rich repeat-containing receptors (NLRs) family pyrin domain containing 3) is one of the best-described inflammasome proteins, and EVs isolated from HNSCC patients, which were enriched in TGF-β signaling molecules, were able to inhibit the induction of pro-IL-1β and pro-caspase-1 proteins, in addition to the downregulation of *NLRP3* expression during the priming phase of inflammasome activation (Bottino et al., 2021). Moreover, MG63 osteosarcoma cell-derived EVs induced M2 macrophage differentiation and also enhanced expression of cytokine transcripts, such as *IL10*, *VEGF* and *TGFB1 in vivo* (Cheng et al., 2021). Nonetheless, although this study claimed that T cell immunoglobulin and mucin domain containing-3 (TIM-3) was the mediator of such effect transferred by osteosarcoma-derived EVs (Table 1), the mechanisms by which this protein drives immunosuppression and *TGFB1* expression were not fully investigated. In the context of TDEs inducing TGF-β1 expression in T cells, nasopharyngeal cancer (NPC)-derived EVs recruited T_reg_ cells, inducing TGF-β1 release, and converting CD4^+^CD25^−^ T cells to CD4^+^CD25^high^ T cells (Mrizak et al., 2015). Moreover, EVs isolated from esophageal cancer (ECA) cells carrying lysosomal associated membrane protein 1 (LAMP1) and MMP-9 induced naive B cells to differentiate into TGF-β-producing regulatory B cells (Table 1), which led to immunosuppressive effects on CD8^+^ T-cells (Li et al., 2015).

Together, all these cases show that EVs derived from different types of tumors are often loaded with TGF-β1, educating the immune system to act in favor of tumorigenesis. Thus, the use of approaches to avoid the delivery of EVs carrying TGF-β1 (e.g. anti-TGF-β1 antibodies or hybrid anti-PD-L1 and TGFβRII biomolecules, such as bintrafusp-α (Gulley et al., 2021)) is a promising tool to avoid the immune evasion promoted by EVs and TGF-β signaling. Nevertheless, it is important to mention that TGF-β signaling is not the only pathway regulated by tumor-derived EVs and acting on immune cells. Hence, to inhibit the immunomodulatory mechanism promoted by EVs, their multi-factorial mode of action remains to be targeted.

### Extracellular Vesicles and Their Cargo as Tumor Biomarkers and Drug Resistance

The management and prognosis of different cancer patients have improved over the last decades. Yet, a significant proportion of patients still fail the treatment protocols incorporating radiotherapy, chemotherapy, targeted therapy and immunotherapy. Biomarker-directed therapeutic decisions remain the cornerstone for precision oncology, most widely practiced when protocols of targeted therapy are utilized. The mechanisms driving treatment resistance in cancer cells are multi-factorial. TGF-β has been implicated as a key player for treatment resistance in several tumors, since TGF-β signaling induces EMT, maintains stem-like cell populations in tumors and modulates the TME (Colak and ten Dijke, 2017). Furthermore, EVs can also mediate drug resistance by *1*) transferring functional proteins and possibly even functional RNAs from resistant donor cells to sensitive recipient cells, *2*) sequestering drugs from the target sites, causing reduction to the local cytotoxic concentration (Samuel et al., 2017), and *3*) by carrying membrane proteins that can capture therapeutic monoclonal antibodies which aimed at blocking target receptors at the tumor cell surface (Whiteside, 2016). Therefore, assuming that the role of TGF-β signaling in controlling the content of EVs as previously suggested (Fricke et al., 2019b) can be strengthened, one can consider that EVs carrying molecules linked to TGF-β signaling can provide new mechanisms to understand not only how resistance to treatment rises in tumors, but also how the resistance can spread through tumor cells that exhibit rather heterogeneous phenotypes.

Thus, one of the first reports connecting TGF-β signaling molecules carried by EVs to resistance to cancer treatment involved HCC (Takahashi et al., 2014). TGF-β significantly induced the expression of several lncRNAs, including the *linc-RoR* (Table 1), which was also enriched in HCC-derived EVs (Takahashi et al., 2014). Mechanistically, *linc-RoR* induced chemoresistance to the protein kinase receptor inhibitor sorafenib, due to an increase in the number of CD133^+^ tumor-initiating cells (Takahashi et al., 2014). However, whether *linc-RoR* could be functionally delivered to the recipient cells by EVs, or whether the chemoresistance phenotype transferred by EVs to HCC cells was due to other EV cargo molecules, affected by the action of *linc-RoR* in the donor cells, remains unresolved. Moreover, PC-associated CAFs release EVs carrying *miR-423-5p*, which targets *gremlin-2* (*GREM2*) (Shan et al., 2020). The downregulation of *GREM2* by *miR-423-5p* increased PC resistance to taxanes (Table 1). GREM2 is a known extracellular ligand-binding inhibitor of bone morphogenetic proteins (BMP), and thus, the proposed mechanism by which GREM2 could impact TGF-β signaling awaits further studies. CRC CAF-derived EVs can deliver the *miR-93-5p* to CRC cells (Chen et al., 2020). Mechanistically, *miR-93-5p* targeted *FOXA1* transcripts (Table 1), inducing their downregulation, and due to the lack of FOXA1, the *TGFB3* gene promoter is de-repressed (Chen et al., 2020). Hence, by increasing TGF-β3 levels, EVs containing *miR-93-5p* and secreted from CRC CAFs, increased CRC tumor growth and rescued these cells from radiation-induced apoptosis (Chen et al., 2020). HNSCC cell lines that exhibit resistance to the widely used chemotherapeutic cisplatin, released EVs carrying TGF-β3 and were capable of transferring the drug-resistant phenotype to sensitive cells through activation of TGF-β signaling (Rodrigues-Junior et al., 2019). Furthermore, this study also evaluated TGF-β3 levels in specific EV fractions circulating in the plasma of HNSCC patients treated with chemo-radiation (Rodrigues-Junior et al., 2019). In this screen, TGF-β3 was significantly more abundant in the plasma EVs of HNSCC patients that did not respond to chemo-radiation treatment and the high levels of TGF-β3 in plasma EVs was associated with poor progression-free survival, highlighting the relevance of the use of EV-based biomarkers in oncology. Moreover, EVs derived from BRCA cells mediated intercellular transfer of TGF-β1 in addition to inducing EMT and increasing resistance to the cytotoxic drug adriamycin (Tan et al., 2021). Hence, since the EV content can reflect features of the tumor that secretes them, studies on EVs may generate important insights into the tumor milieu, with potential to identify reliable markers not only for prognosis but also for cancer detection and subtype segregation (Rodrigues-Junior et al., 2019; Hoshino et al., 2020).

Anti-cancer immunotherapies aiming at reviving tumor-reactive CTLs are promising in a large group of metastatic patients, but immune evasion can develop. Hence, by understanding the molecular mechanisms of resistance to immunotherapy, better strategies can be adopted to improve the clinical outcome for cancer patients (Sharma et al., 2017). Thus, immunosuppressive molecules, including programmed death-ligand 1 (PD-L1) and TGF-β1 (Table 1), which facilitate tumor immune evasion, are carried by tumor-derived EVs (Mathew et al., 2020). Beyond PD-L1 and TGF-β, cancer-derived EVs may act as immune system suppressors based on the mechanism of NKG2D downregulation and subsequent natural killer cell cytotoxicity suppression described above (Szczepanski et al., 2011). Alternatively, cargoes such as miRNAs or enzymatically active arginase-1, can impact on macrophage differentiation or T cell activation respectively (reviewed in (Zhou et al., 2020; Marar et al., 2021)). Of note, when human lung fibroblasts are stimulated with TGF-β1, activate SMAD2/3 and YAP/TAZ signaling, which enhance the deposition of PD-L1 into EVs (Kang et al., 2020). Similarly, TGF-β present in the TME can induce BRCA cells to release EVs loaded with PD-L1, while the blockage of TGF-β signaling by a chemical inhibitor (SB431542) reduced the PD-L1 levels of these EVs (Chatterjee et al., 2021). Furthermore, when PD-L1 is present on the surface of EVs that circulate systemically, this ligand can bind to its receptor PD-1 on effector T cells, eliciting the immune checkpoint response (Chen et al., 2018; Poggio et al., 2019; Kang et al., 2020). Therefore, the levels of PD-L1 on EVs cannot only stratify clinically tumor patients treated with anti-PD-1 antibodies as responders and non-responders, but also represent a new therapeutic target, that may possibly overcome resistance to current anti-PD-1 immunotherapies (Chen et al., 2018; Theodoraki et al., 2018; Poggio et al., 2019). Furthermore, a phase I/II trial for clinical markers in glioma patients receiving anti-tumor vaccines evaluated the mRNA content of circulating EVs in the plasma of patients pre- and post-vaccine treatment (Muller et al., 2015). In this trial, *TGFB1* and *IL-8* mRNA positively correlated with the immunologic responses to glioma antigens in the EVs isolated from the post-vaccine group, suggesting the potential of mRNAs carried by EVs to assess the response of vaccination therapy in glioma patients (Muller et al., 2015). It is worth noting that such studies aimed at biomarker discovery can be valid even in the case of monitoring fragmented RNAs in the EV cargo.

One of the largest screens to identify TDE proteins as new biomarkers for early-stage cancer detection and identification of primary tumor types in patients of many different cancers was recently conducted (Hoshino et al., 2020). The analysis of TDEs in this study identified the ECM or transmembrane proteins thrombospondin 2 (THBS2), versican (VCAN), and tenascin C (TNC) as genes whose expression analysis could distinguish tumors from normal patients with 90% sensitivity and 94% specificity (Hoshino et al., 2020). Both VCAN and TNC are upregulated by TGF-β signaling (Jinnin et al., 2004; Yeung et al., 2013), whereas thrombospondins regulate latent TGF-β activation in the ECM (Munger et al., 1997). Of note, fibronectin, another protein upregulated by TGF-β signaling, was found highly expressed in all (426 human tissue explants, plasma and other bodily fluids) TDEs evaluated in this study (Hoshino et al., 2020). In another screen of EV proteins aiming at revealing new biomarkers for PC patients, using reverse-phase protein microarrays, a protein signature with prognostic significance was identified, with TGF-β1 being among the proteins presented by EVs with statistical significance in recurrent PC patients (Signore et al., 2021).

Screening for miRNAs circulating in the plasma of pancreatic cancer patients, a signature of six miRNAs that was able to distinguish non-tumor from cancer tissue was reported (Zhou X. et al., 2018). One of the six miRNAs, the *miR-122-5p*, was also enriched in EVs circulating in the plasma of the patients in comparison to non-cancer patients (Zhou X. et al., 2018). Moreover, *in silico* data showed that *miR-122-5p* could have a relevant role in negatively regulating TGF-β signaling by targeting *TGFBR2* transcripts (Ding et al., 2020). A screen for lncRNAs upregulated by TGF-β in pancreatic cancer that could enhance cargo levels loaded into PDAC-derived EVs, identified 21 such lncRNAs including *HULC* (Takahashi et al., 2020). The level of *HULC* in PDAC-derived EVs was further validated by digital PCR as significantly increased in PDAC patients compared to healthy individuals or intraductal papillary mucinous neoplasm patients, suggesting that non-coding RNAs regulated by TGF-β can contribute to the diagnosis for human PDAC. Hence, the evidence suggests that the molecular content of EVs is a new promising tool that can allow oncologists to improve early tumor detection and offer treatment decisions to cancer patients (Rodrigues-Junior et al., 2019; Hoshino et al., 2020; Signore et al., 2021). Furthermore, large-scale production of EVs from healthy cells, such as MSCs, may be an alternative avenue to deliver promising molecules that may facilitate multiple translational approaches, including the fight against resistance to cancer therapy (Witwer et al., 2019).

## Conclusion

This article aims primarily at providing a comprehensive view of the relationship between EVs and TGF-β in the context of cancer (Figure 2). This relationship can be summarized as follows: *1*) TGF-β signaling regulates the enrichment of specific cargo molecules (proteins, RNAs, metabolites) in EVs, thus offering a qualitative input for downstream functions mediated by the EVs. The large number of examples presented in the article fall into this category, and in this manner, TGF-β signaling joins many other growth factor and cytokine pathways that regulate the content of EVs. Yet, the functional significance of RNA cargo molecules must be viewed with caution. *2*) EVs carry as cargo ligands of the TGF-β family or other key components of the TGF-β signaling machinery, possibly including their respective mRNAs, as evidenced in a variety of cancers. Thus, recipient cells in various tissues, including the TME and the pre-metastatic niche respond to TGF-β, usually mediating pro-tumorigenic actions. *3*) EVs carry indirect regulators of TGF-β signaling, possibly including miRNAs that target key signaling components (e.g. the receptors) or lncRNAs that sponge complementary miRNAs and thus relieve TGF-β signaling from negative regulators. These three scenarios provide an elaborate series of mechanisms by which TGF-β as a component of EVs, and EVs, as cell-to-cell communication vehicles, coordinate processes critical for cancer development. Since TGF-β, like many other cytokines, is known to be secreted and deposited in the ECM of tumors, its presence as EV cargo and that of its various regulators leaves the interested investigator with the central question of what might the purpose of using such alternative routes of TGF-β delivery to recipient cells in the TME be. Is TGF-β cargo in EVs biologically different from TGF-β deposited in the ECM? Is the distance between tumor cells that produce TGF-β and tumor or stromal cells that respond to TGF-β a decisive factor that necessitates transport *via* EVs instead of local ECM deposition? These central questions remain open for investigation as, at the same time, the link between TGF-β and EV biology promises important contributions to biomarker and novel treatment development in several, if not all, cancer types.
